# High TB burden and low notification rates in the Philippines: The 2016 national TB prevalence survey

**DOI:** 10.1371/journal.pone.0252240

**Published:** 2021-06-04

**Authors:** Mary Ann D. Lansang, Marissa M. Alejandria, Irwin Law, Noel R. Juban, Maria Lourdes E. Amarillo, Olivia T. Sison, Jose Rene B. Cruz, Concepcion F. Ang, Joseph Adrian L. Buensalido, Johanna Patricia A. Cañal, Nina T. Castillo-Carandang, Cynthia P. Cordero, Donna Mae G. Gaviola, Mary Ann J. Ladia, Jacinto Blas V. Mantaring, Myrna T. Mendoza, Maria Sonia S. Salamat, Hilton Y. Lam, Marina Tadolini, Anna Marie Celina G. Garfin, Philippine NTPS 2016 Group

**Affiliations:** 1 Department of Clinical Epidemiology, College of Medicine, University of the Philippines, Manila, Philippines; 2 Division of Infectious Diseases, Department of Medicine, College of Medicine, University of the Philippines–Philippine General Hospital, Manila, Philippines; 3 Global Tuberculosis Programme, World Health Organization, Geneva, Switzerland; 4 Institute of Clinical Epidemiology, National Institutes of Health, University of the Philippines, Manila, Philippines; 5 Foundation for the Advancement of Clinical Epidemiology, Manila, Philippines; 6 Social Innovation in Health Initiative, University of the Philippines, Manila, Philippines; 7 Foundation for the Control of Infectious Diseases, Taft Avenue, Manila, Philippines; 8 Department of Radiology, University of the Philippines College of Medicine, University of the Philippines, Manila, Philippines; 9 Philippine College of Radiology, Quezon City, Philippines; 10 Department of Health, National TB Control Program, Manila, Philippines; 11 Institute of Health Policy and Development Studies, University of the Philippines, Manila, Philippines; 12 Infectious Diseases Unit, IRCCS Azienda Ospedaliero-Universitaria di Bologna, Policlinico di Sant’Orsola, Bologna, Italy; 13 Department of Medical and Surgical Sciences, Alma Mater Studiorum University of Bologna, Bologna, Italy; The University of Georgia, UNITED STATES

## Abstract

**Setting:**

The 3^rd^ national tuberculosis (TB) survey in the Philippines in 2007 reported a significant decline in the prevalence of TB. Since then, more significant investments for TB control have been made, yet TB burden estimates from routine surveillance data remain relatively stable.

**Objective:**

To estimate the prevalence of bacteriologically confirmed pulmonary TB in the Philippines amongst individuals aged ≥15 years in 2016.

**Design:**

In March–December 2016, we conducted a population-based survey with stratified, multi-stage cluster sampling of residents in 106 clusters aged ≥15 years. Survey participants were screened for TB by symptom-based interview and digital chest X-ray. Those with cough ≥2 weeks and/or haemoptysis and/or chest X-ray suggestive of TB were requested to submit 2 sputum specimens for Xpert MTB/RIF, direct sputum smear microscopy using LED fluorescent microscopy, and mycobacterial solid culture (Ogawa method). Bacteriologically confirmed pulmonary TB was defined as MTB culture positive and/or Xpert positive.

**Results:**

There were 46,689 individuals interviewed, and 41,444 (88.8%) consented to a chest X-ray. There were 18,597 (39.8%) eligible for sputum examination and 16,242 (87.3%) submitted at least one specimen. Out of 16,058 sputum-eligible participants, 183 (1.1%) were smear-positive. There were 466 bacteriologically confirmed TB cases: 238 (51.1%) Xpert positive, 69 (14.8%) culture positive, and 159 (34.1%) positive by both Xpert and culture. The estimated TB prevalence per 100,000 population aged ≥15 years was 434 (95% CI: 350−518) for smear-positive TB, and 1,159 (95% CI: 1,016−1,301) for bacteriologically confirmed TB.

**Conclusion:**

This nationally representative survey found that the TB burden in the Philippines in 2016 was higher than estimated from routine TB surveillance data. There was no evidence of a decline in smear and culture positive TB from the 2007 survey despite significant investments in TB control. New strategies for case-finding and patient-centered care must be intensified and expanded.

## Introduction

Globally, tuberculosis (TB) ranked 10th amongst the leading causes of death worldwide and is the leading cause from a single infectious agent with an estimated 1.2 million TB deaths amongst HIV-negative people and 10 million new cases of TB in 2019 [1]. Globally, the Philippines remains one of the highest TB burden countries and continues to bear the health and socioeconomic consequences of TB. In 2015, TB ranked 4^th^ amongst the leading causes of mortality in the country and 9^th^ in terms of disability-adjusted life years [2]. The estimated incidence of TB was 322 per 100,000, while the estimated number of multi-drug resistant TB cases amongst notified pulmonary TB cases was 15,000 for 2015 [3].

National TB prevalence surveys were conducted in 1981–83, 1997, and 2007 [4–6]. The 2007 survey reported a 31% reduction in bacteriologically positive TB and 27% reduction of smear-positive TB prevalence compared to the 1997 survey, attributed mainly to the implementation, and roll out of the Directly Observed Treatment, Short course (DOTS) program in the late 1990s and early 2000s, complemented by strategic public-private partnerships. The 2007 survey concluded that TB was still a major public health problem in the Philippines, with a prevalence of 2.6 per 1,000 smear-positive TB and 6.6 per 1,000 bacteriologically confirmed TB amongst those aged ≥ 10 years [6]. Subsequently, the Government of the Philippines developed a strategic framework for TB control, the Philippine Plan of Action to Control Tuberculosis (2010–2016), as part of its health sector agenda. The implementation of the national strategy involved an increased budget for the National TB Control Program (NTP), expansion of NTP priorities from smear-positive TB cases to all forms of TB, engagement of village health workers and communities in case detection and treatment activities, expansion of private sector and public hospital engagement in DOTS programs, and leveraging funding from the Global Fund to Fight AIDS, Tuberculosis and Malaria to expand treatment centers for multidrug-resistant TB (MDR-TB) [7].

The NTP of the Philippines’ Department of Health (DOH) needed to reassess the TB burden and identify the gaps and challenges whilst shifting the TB target of the Millennium Development Goals −a decline in TB incidence by 2015 − to that of the Sustainable Development Goals − 80 per cent reduction in TB incidence compared to 2015 [8]. Hence, we conducted the 2016 National TB Prevalence Survey to provide the NTP with accurate and updated information on TB burden as the Philippines segues into its 2017−2022 strategic plan to eliminate TB.

The primary objectives of the survey were to estimate the prevalence of bacteriologically confirmed pulmonary TB (PTB) amongst the population in the Philippines ≥15 years during 2016, and to assess the trends of smear-positive and bacteriologically confirmed PTB prevalence, compared with the results of the 2007 survey.

## Study population and methods

### Study design, sample size and sampling procedure

We conducted a population-based, cross-sectional survey from March to December 2016 across 57 provinces from 17 regions of the Philippines. We designed the survey in accordance with the WHO Global Task Force on TB Impact Measurement [9].

The sample size was calculated using the following assumptions: (1) a maximum 20% reduction in smear-positive TB since 2007, therefore prevalence of smear-positive TB was assumed to be 2.6 per 1,000 individuals aged ≥15 years in 2016; (2) cluster size of 500 individuals eligible to attend the survey; (3) coefficient of between-cluster variation of 0.8 with a corresponding design effect of 1.83; (4) 25% relative precision; and (5) 85% minimum participation rate. The estimated sample size was 51,000 eligible individuals from 102 clusters. Six clusters were added to round off allocations to strata and to account for the cancellation of clusters due to security reasons and/or inaccessible areas, to give a final sample size of 54,000.

**Fig 1:**
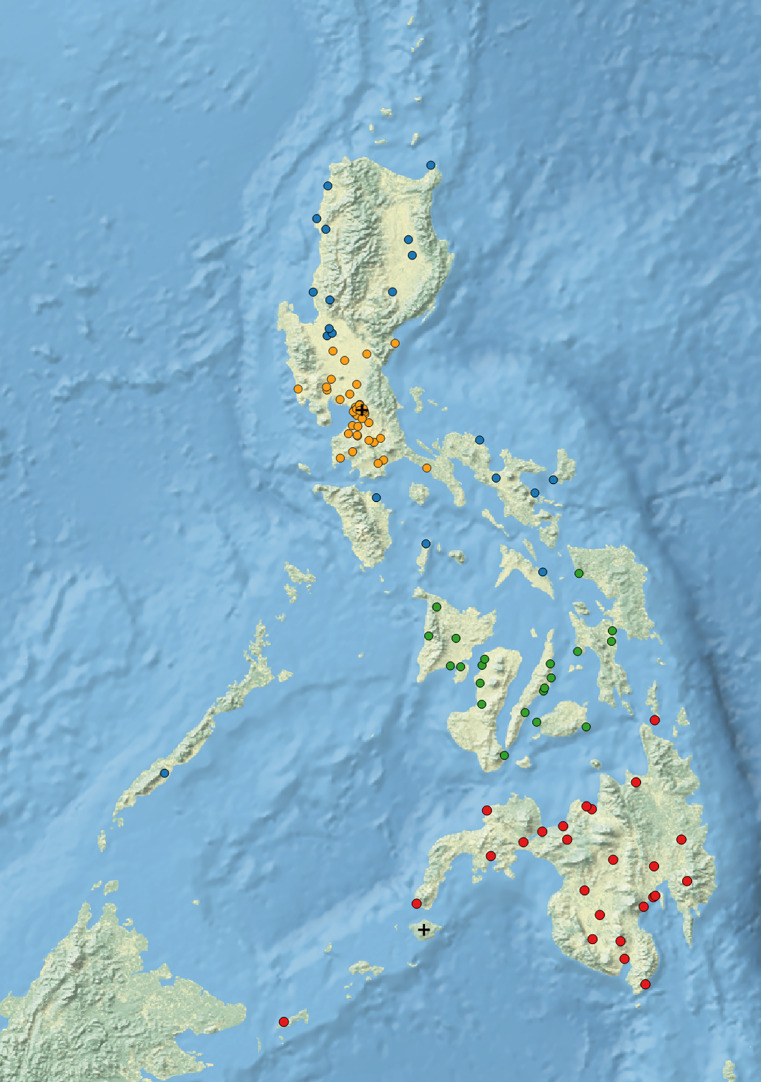
Distribution of the survey clusters across the Philippines, NTPS 2016 (N = 106 clusters). Orange dots–stratum 1 (National Capital Region; blue dots–stratum 2 (rest of Luzon island); green dots–stratum 3 (Visayas region); red dots–stratum 4 (Mindanao region); cross marks (+) − sampled clusters that were dropped.

Using stratified, multi-stage cluster sampling, 108 clusters were randomly sampled across 4 strata, allocated proportionately to population size as follows: 41 clusters for stratum 1 − National Capital Region, Central Luzon region, and Calabarzon region; 20 for stratum 2 − rest of Luzon island; 21 for stratum 3 − Visayas region; and 26 for stratum 4 − Mindanao region (Fig 1).

### Survey procedure

Four survey teams were deployed simultaneously to cover each stratum. Individuals aged ≥15 years old and residing in the cluster for at least 2 weeks before the survey who provided informed consent were interviewed using a structured questionnaire with the following components: (1) TB symptoms screening (cough and duration, sputum production, haemoptysis, fever, weight loss, night sweats); (2) past and/or current TB diagnosis and treatment; (3) health-care seeking behavior of those reporting symptoms; and (4) a history of diabetes mellitus and smoking. After the interview, all participants who consented underwent chest X-ray using a mobile digital machine (MinXray HF 120/60H Powerplus^TM^), except for pregnant women, those with physical disabilities that precluded upright chest X-ray, a recent chest X-ray, and, in a few instances, X-ray machine malfunction. A trained physician (*i*.*e*., field team leader) further interviewed and examined the participants, then interpreted their chest X-rays at the survey site. The digital images were then sent to an off-site radiologist within the day. The off-site radiologists used the following classification, adapted from the WHO TB prevalence survey handbook [8]: (1) normal chest X-ray; (2) abnormality detected–not TB; (3) pulmonary abnormality detected–not TB; and (4) pulmonary abnormality detected–suspicious for TB.

### Eligibility for sputum examination

Participants with screening symptoms of cough ≥2 weeks and/or haemoptysis, and/or radiographic abnormalities suggestive of TB based on the reading of the field team leader were requested to submit 2 sputum specimens: (1) a spot specimen at the survey site, and (2) an early morning specimen collected the next day. Those who did not have a chest X-ray were also instructed to submit sputum, irrespective of screening symptoms. If the volume of the sputum specimens was <3 ml, a third specimen was collected. Participants with discordant readings of their chest X-rays, i.e., normal reading by the field reader and suggestive of TB by the offsite radiologist, were recalled to submit sputum.

### Laboratory procedures

The sputum specimens from the field sites were in tightly sealed containers and packaged in insulated ice boxes at 2–8°C with temperature monitor, and transported at least twice a week to the designated six reference laboratories located in the three major island groups of the Philippines. Xpert MTB/RIF version G4 assay (Xpert®) was done on the spot sputum specimens while direct sputum smear microscopy (DSSM) using light emitting diode fluorescence microscope (LED-FM) and mycobacterial culture on Ogawa solid media (one tube) were performed, preferably on the morning specimens, and incubated and observed for growth up to 8 weeks. Tubes with colony growth were sampled, smeared, and stained by Ziehl Neelsen stain, and acid-fast bacilli (AFB) subjected to the MPT64 rapid test for MTB species confirmation. LED-FM readings of sputum smears were reported using the NTP Manual of Procedures scale for AFB microscopy [10], ranging from 0 (no acid-fast bacilli), scanty, 1+, 2+, and 3+ (>250 AFB in one field on average at 200x magnification or >60 AFB at 400x magnification). Isolates from MTB culture were forwarded to the Research Institute for Tropical Medicine–National TB Reference Laboratory of the Philippine Department of Health for drug sensitivity testing (DST), using the proportion method in 92% and line probe assay in 8% of the isolates.

### Survey case definitions

Bacteriologically confirmed TB (BCTB) cases were defined as screen-positive participants with sputum samples positive for MTB on culture and/or Xpert MTB/RIF positive. The Diagnostic and Medical Panel, composed of pulmonologists, infectious disease specialists, a senior radiologist, the laboratory coordinator, one data manager, and the deputy director of the Central Technical Unit, deliberated on the survey classification of all participants with any positive results either on DSSM, Xpert, or culture (S1 Table).

### Quality management procedures

The quality management unit monitored the field teams and participating laboratories to ensure that survey and laboratory processes adhered to the project standard operating procedures. Senior radiologists at the central level reviewed all chest X-rays of participants with positive laboratory results, all abnormal chest X-rays, and chest X-rays with discordant readings between the field team leader and the off-site radiologist, and a random sample of 5% of normal chest X-rays. External technical assistance was provided by members of the WHO Global Task Force on TB Impact Measurement, the WHO Country Office, and the WHO Regional Office.

### Data management and analysis

Survey teams captured data via tablet computers. Barcodes were used to identify survey participants, including their sputum specimens and laboratory results, stored in a non-relational database management system. Data entry and data checking programmes were developed using Epi Info^TM^ versions 3.5.4 and 7.1 [11]. The central data management unit cleaned, validated, and merged all electronic databases from the field teams, reference laboratories, off-site radiologists, and central radiologists.

STATA® version 14 was used for data analysis (StataCorp, Texas, USA). We used the best-practice analytical methods recommended by the WHO Global Task Force on prevalence surveys to estimate TB prevalence to account for cluster sampling, non-participation, and missing data [9, 12]. Three logistic regression models were conducted: (1) cluster-level analysis, (2) individual-level analysis, and (3) estimation with inverse probability weighting and with multiple value imputation. Only model 3 is presented in this article. To determine possible risk factors for TB, multiple logistic regression analysis with inverse probability weight and backward elimination strategy was done.

As an approximate indicator of case detection [13], we also estimated the prevalence to case notification ratios (P:N) by comparing the age- and sex-specific BCTB prevalence rates to TB case notification rates for smear-positive PTB for the same age groups and sex as reported in the national TB registry for 2016. Lastly, we compared estimated prevalence rates for smear-positive and culture-positive TB in the 2007 and 2016 national TB prevalence surveys (NTPS). Recognizing that there were fundamental differences between the two surveys in the sampling design, age groups covered, screening procedures, chest X-ray imaging interpretation, diagnostic tests used, and TB case definition, we restricted our comparison of the two surveys to the following common parameters: age eligibility, screening outcomes defined by chest X-ray only, and PTB cases defined by Ogawa culture only. This allowed estimations of adjusted prevalence rates for 2007 and 2016 for culture-positive TB cases as well as smear-positive/culture-positive TB.

### Ethics statement

On January 19, 2016, the National Ethics Committee approved the survey protocol, standard operating procedures, data collection forms, and consent forms in English and four Philippine languages. The study was done in accordance with the National Ethical Guidelines for Health and Health Related Research and the Data Privacy Act of 2012. The written informed consent forms were supplemented with an individually administered video information using a tablet computer, outlining the survey procedures as well as the risks and benefits of participation. Minors (below 18 years) provided written assent, but their parent or guardian also provided written informed consent. A two-item quiz was administered to document competence and comprehension of the participants.

The city/municipal health officer and the NTP coordinator per cluster were informed of participants who were classified as TB cases in their respective areas via password-protected email communications. Clinical recommendations on management for each TB case including the x-ray and laboratory results were also provided to the health officers by the Diagnostic and Medical Panel.

## Results

### Survey participants

**Fig 2:**
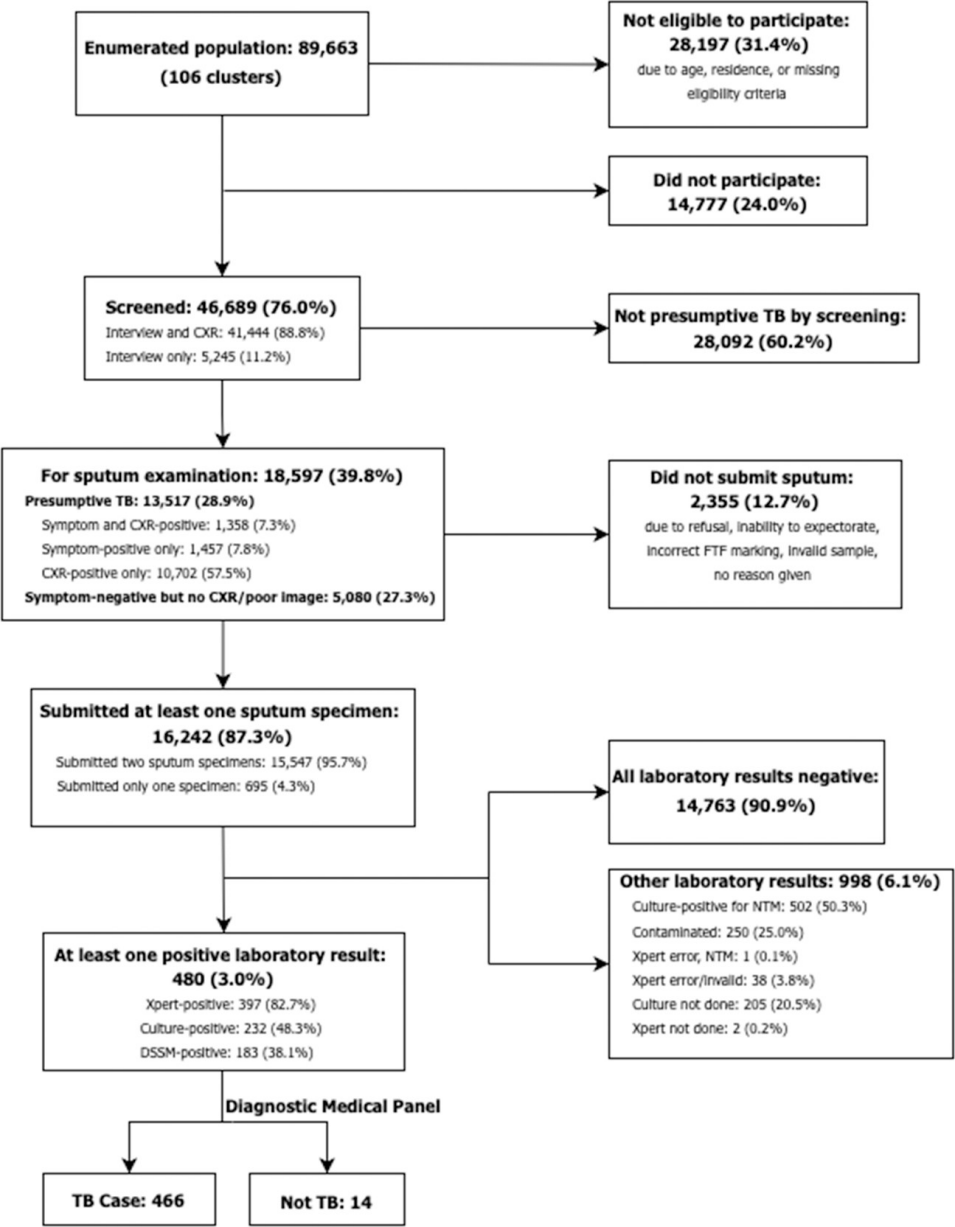
Flow diagram of procedures in the 2016 TB prevalence survey in the Philippines (n = 106 clusters). (1) CXR: Chest X-ray; FTF: Field tracking form; DSSM: Direct sputum smear microscopy. (2) One sputum: no laboratory result (error on two Xpert runs; no DSSM and MTB culture).

There were 89,663 individuals enumerated in106 clusters of the 108 original clusters in the sampling frame. Two clusters were eventually dropped–one due to safety issues in a conflict-affected area and the other due to an inability to do household enumeration and community mobilization due to restrictions imposed by the village officers. Compared with the 2010 Philippine census, the age and sex distributions of the population in the clusters were similar in the survey-eligible population, except for slightly higher proportions (1.5–1.6%) in the age groups aged ≥55 years in the 2016 NTPS enumeration, for which we did not make population-adjusted estimates. Of the total enumerated, 28,197 (31.4%) were not eligible to participate due to age or residence criteria. Of those eligible to participate, 46,689 (76.0%) attended the survey, resulting in an average of 440 participants per cluster (range: 149–572). Participation was significantly higher amongst women (82.2%) than men (69.5%); individuals aged ≥65 years (90.1%) than those aged ≤25 years (70.0%); and amongst those in rural clusters (80.2%) than in the urban clusters (71.5%) (all *P*<0.001) (Fig 2).

### Screening

Amongst participants, 46,689 (100%) were screened for symptoms and 41,444 (88.8%) underwent chest X-ray. There were 18,597 (39.8%) who were assessed as eligible for sputum collection, out of whom 10,702 (57.5%) had chest X-ray abnormalities suggestive of TB alone; while 1,457 (7.8%) had screening symptoms alone and 1,358 (7.3%) were positive on both chest X-ray and screening symptoms. Another 5,080 (27.3%) who did not report symptoms refused or were unable to undergo chest X-ray (Fig 2).

### Laboratory results

Of the 18,597 eligible for sputum specimen collection, 16,242 (87.3%) submitted at least one sputum sample, while 15,547 (83.6%) submitted two samples. Laboratory results were available for 16,241 (99.9%) samples. A total of 16,058 (98.9%) had a valid DSSM, out of which 183 (1.1%) were smear-positive. There were 16,200 (99.7%) with valid Xpert results, out of which 397 (2.4%) were Xpert-positive. Valid culture results were available for 15,776 (97.1%), out of which 232 (1.5%) were MTB-positive (Fig 2). Of the 232 MTB-positive samples, there were 4 weakly positive samples (≤9 colonies) that were both smear-negative and Xpert-negative.

### Survey TB cases

**Fig 3:**
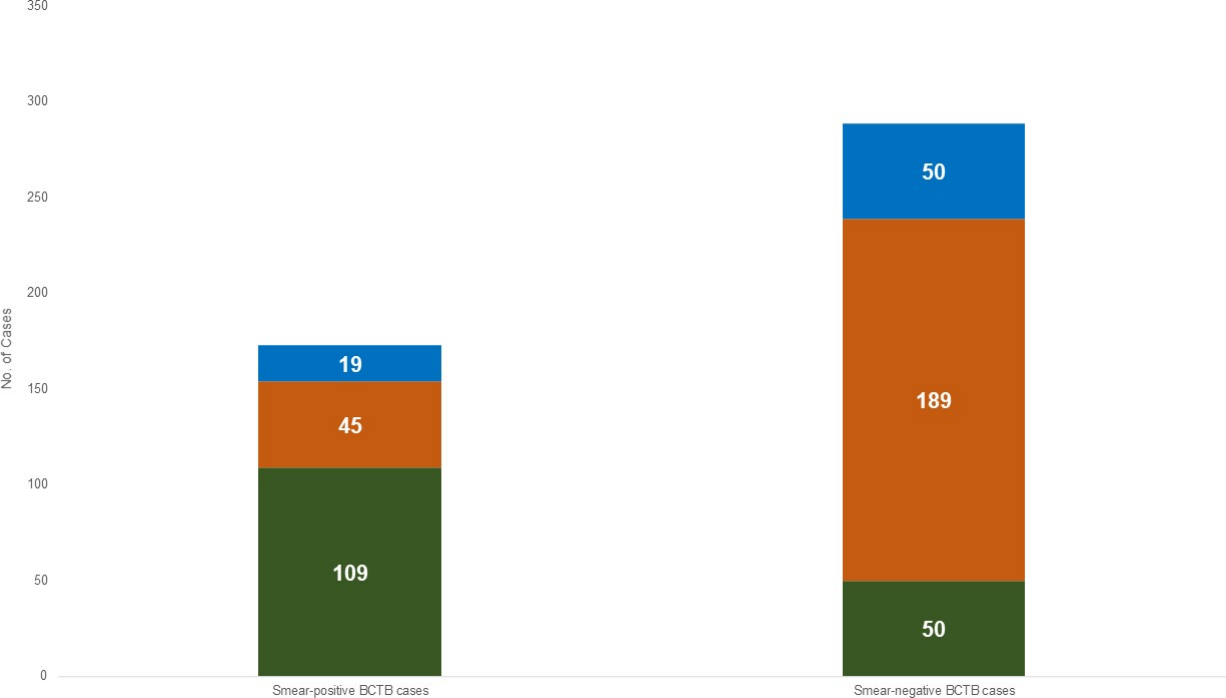
Classification of bacteriologically confirmed TB cases by smear, (n = 466). Green bars − culture-positive and Xpert-positive; orange bars − culture-negative and Xpert-positive; blue bars − culture-positive and Xpert negative. BCTB: bacteriologically confirmed TB.

There were 466 bacteriologically confirmed pulmonary TB cases, only 173 (37.1%) of whom were smear-positive. Based on the survey case definition using Xpert and/or MTB culture, 238 (51.1%) were Xpert-positive only, 69 (14.8%) were culture-positive only, and 159 (34.1%) were both Xpert and culture-positive (Fig 3).

Regarding the extent of TB recurrence among the 466 survey TB cases, 77 (16.5%) reported previous TB treatment at some point in the past five years and 30 (6.4%) stated they were on treatment at the time of the survey. Thus, 359 (77%) were considered new cases. Amongst the 238 survey TB cases who were positive by Xpert alone, 71 (29.8%) had current or previous history of TB treatment, compared to 36 out of 228 (15.8%) who were positive by culture alone or Xpert with culture. Rifampicin resistance was detected in 29 (7.3%) of the 397 Xpert-positive cases, with a significantly higher proportion seen among those with previous TB treatment (15 out of 81, 18.5%) compared to the treatment-naive cases (14 out of 385, 3.6%). Three of the 29 rifampicin-resistant specimens detected by Xpert were rifampicin-susceptible by DST. Overall, rifampicin resistance was 5.7% by DST (13 isolates). Out of 229 available MTB isolates, resistance to one or more anti-TB drugs were as follows: isoniazid alone − 5.7% (13 isolates); streptomycin alone − 3.0% (7 isolates); isoniazid and streptomycin– 2.6% (6 isolates); rifampicin and isoniazid (MDR-TB), with or without other first-line or second-line drugs −3.9% (9 isolates).

Only 150 (32.1%) of the BCTB cases reported screening symptoms, with symptoms being more frequent among smear-positive TB cases (88 out of 173, 50.9%) compared to smear-negative TB cases. On the other hand, the frequency of screening symptoms among the sputum-eligible participants who were non-cases was much lower (2,537 out of 15,775, 16.1%).

Among BCTB cases with screening symptoms, cough of ≥2 weeks duration was the most frequently reported symptom among the BCTB cases (n = 117, 25.1%), followed by both cough of ≥2 weeks and haemoptysis (n = 22, 4.7%), while haemoptysis alone was present in only 5 cases (1.1%). The proportion of BCTB cases reporting cough of ≥2 weeks was significantly higher than among non-cases (P<0.001) (Table 1).

**Table 1:** Comparison of self-reported symptoms in survey cases and non-cases among the sputum-eligible survey participants, 2016 NTPS, Philippines (n = 16,241).

Symptoms	Survey cases (n = 466) No. (%)	Non-cases^a^ (n = 15,775) No. (%)
**With screening symptoms**	**150 (32.2)**	**2,543 (16.1)**
Cough ≥14 days only	117 (25.1)	2,036 (12.9)
Haemoptysis only	5 (1.1)	188 (1.2)
Cough ≥14 days & haemoptysis	22 (4.7)	176 (1.1)
Cough <14 days & haemoptysis	6 (1.3)	137 (0.9)
With screening symptoms, unspecified	0 (0.0)	6 (0.0)
**Other symptoms**	**195 (41.8)**	**5,636 (35.7)**
Cough <14 days^b^	124 (26.6)	2,802 (17.8)
Other non-specific symptoms^c^	71 (15.2)	2,834 (18.0)
**No symptoms**	**121 (26.0)**	**7,596 (48.1)**

^a^ Non-cases among those sputum-eligible participants.

^b^ With or without symptoms of weight loss, fever, and/or night sweats.

^c^ Symptoms may include weight loss, fever, and/or night sweats.

With regard to chest X-ray screening, 409 out of 438 (93.4%) BCTB cases who underwent chest imaging had radiologic findings suggestive of TB, a yield higher than symptom screening. Notably, two-thirds of cases with X-ray findings were asymptomatic, and these X-ray findings tended to be extensive. Bilateral lung involvement was seen in 275 (67.2%) cases. Ground glass or reticular densities were found in 396 (90.4%) cases–with or without cavities, nodularities, or calcifications. Another 6 had only cavity formation and no other findings.

### Prevalence of TB

The weighted and adjusted prevalence of bacteriologically confirmed pulmonary TB was 1,159 per 100,000 population aged ≥15 years (95% CI: 1,016–1,301) and 434 (95% CI: 350–518) per 100,000 population for smear-positive pulmonary TB. The estimated prevalence rate for culture-positive pulmonary TB was 587 per 100,000 (95% CI: 488 −687). Men were 2.5 times more likely to have pulmonary TB compared to women. The estimated prevalence increased with age, peaking at 45–54 years (1,714 per 100,000 [95% CI: 1,364–2,064]) (Table 2).

**Table 2:** Estimated prevalence of smear-positive, and bacteriologically confirmed pulmonary TB among individuals aged ≥15 years, by sex and age group, 2016 NTPS, Philippines (N = 46,689).

	Smear-positive PTB^a^			Bacteriologically confirmed PTB^a^		
	(N = 173)			(N = 466)		
	No.	Prevalence estimate per 100,000^b^	95% CI	No.	Prevalence estimate per 100,000^a^	95% CI
National	173	434	350–518	466	1,159	1,016–1,301
**Sex**						
Male	125	673	528–819	322	1,713	1,482–1,943
Female	48	205	141–270	144	627	516–739
**Age group (in years)**						
15–24	30	330	197–463	72	799	586–1,011
25–34	24	326	195–458	68	900	677–1,123
35–44	32	470	298–641	76	1,126	821–1,430
45–54	40	665	438–891	103	1,714	1,364–2,064
55–64	24	488	285–691	73	1,504	1,104–1,903
≥65	23	503	310–696	74	1,659	1,261–2,058
**Stratum**						
1	81	599	451−747	187	1,358	1,103−1,612
2	19	258	138−378	77	1,038	787−1,288
3	44	471	261−680	111	1,234	873−1,594
4	29	268	173−364	91	856	686−1,026

^a^ PTB: pulmonary TB.

^b^ Computed using method 3 (multiple value imputation and inverse probability weighting).

Within the age group 15 − 24 years, the weighted prevalence rates for smear-positive TB and BCTB in the 15–19 year old population were 151 per 100,000 (95% CI: 48–254) and 613 (95% CI: 403–822), respectively, suggesting a significant TB burden even among adolescents. There was no significant difference in the estimated prevalence rates among the four geographic strata, but the highest prevalence rate of BCTB was seen in Stratum 1 (1,358 per 100,000 [95% CI: 1,103–1,612]), which has the highest population density in the country at 20,785 persons per square kilometer [14] and includes large settlements of urban slum dwellings.

The estimated prevalence-to-notification (P:N) ratio across all age groups was 3.1, and was higher in men than women. The highest ratio of 4.2 was found in the age group 15–24 years but consistently high throughout (Table 3).

**Table 3:** Prevalence-to-case notification (P:N) ratios, 2016 NTPS, Philippines^a^.

Category	TB case notification rates per 100,000	Estimated prevalence rates per 100,000, 2016 NTPS	P:N ratio
Total ≥15 years	142	434	3.1
**Sex**			
Male	203	673	3.3
Female	82	205	2.5
**Age group (in years)**			
15−24	78	330	4.2
25−34	116	326	2.8
35−44	155	470	3.0
45−54	204	665	3.3
55−64	232	488	2.1
≥65	209	503	2.4

^**a**^ Notification rates were estimated using smear-positive pulmonary TB notifications (2016) obtained from the National TB Control Programme, and population estimates from the UN Population Division (2015 revision).

In the restricted analysis on common survey parameters for 2007 and 2016, the estimated adjusted prevalence rates for culture-positive TB in the adult population were: 463 per 100,000 (95% range: 333−592) and 512 per 100,000 (95% range: 420−603), respectively. For smear-positive culture-positive TB, the estimated adjusted prevalence rates were: 193 per 100,000 (95% range: 117−269) and 286 per 100,000 (95% range: 223−349) for the 2007 and 2016 surveys, respectively.

### Health-care seeking behaviour of symptomatic participants

Of the 46,689 survey participants, only 2,815 (6.0%) reported having screening symptoms. Of these, only 534 (19.0%) consulted a healthcare worker, with more women (271/1,162, 23.3%) seeking care compared to men (263/1,653, 15.9%) (p<0.001). The proportion of participants aged ≥65 years seeking care was significantly higher (164/591, 27.7%, p<0.001) compared to other age groups. No statistically significant difference was seen in the proportion seeking care between the urban (225/1,272, 17.7%) and rural areas (309/1,543, 20%). Amongst those who sought formal care, there were twice more who went to public facilities (359, 12.8%) compared to private providers (166, 5.9%); while only a few went to traditional healers (10, 0.4%). The rest of the participants either did not take any action (1,143, 40.6%) or self-medicated (1,130, 40.1%). Amongst 1,653 men and 1,162 women with screening symptoms, more women (502, 43.2%) self-medicated compared to men (628, 38.0%) (p = 0.005), while more men (759, 45.9%) than women (384, 33.0%) (p<0.001) did not take action. Out of 2,273 survey participants who provided ≥1 reason/s for non-consultation, perceived triviality of symptoms (937, 41.2%), access issues like cost of travel and distance to the health facility (875, 38.5%), and concern about missing school or work (169, 7.4%) were the main reasons for not seeking formal health care.

**Fig 4:**
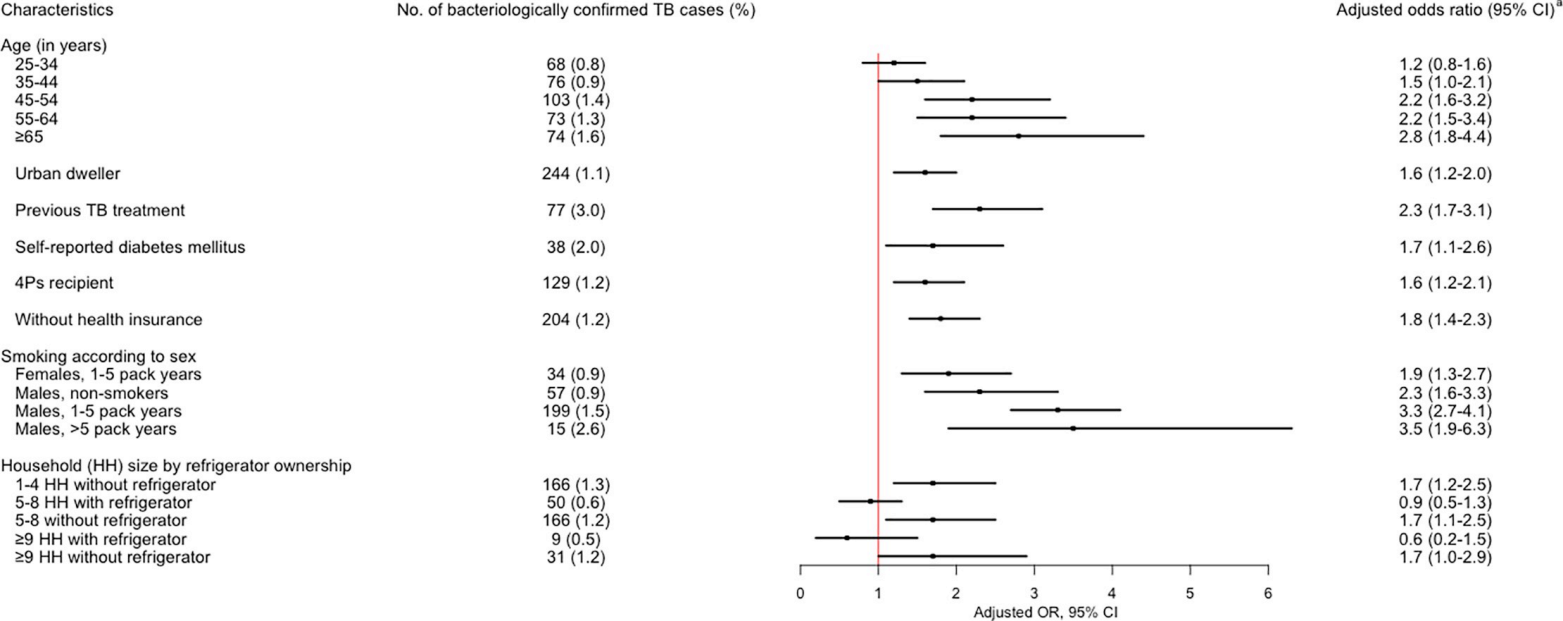
Risk factors for pulmonary TB among survey participants (N = 46,689)^a^. ^a^Adjusted odds ratios (aOR) were estimated using survey logistic regression with stratified cluster design and adjusted for inverse probability weights. 4Ps: *Pantawid Pamilyang Pilipino Program*, a cash transfer program for the poor.

Fig 4 shows the risk factors independently associated with BCTB on multivariable analysis. Age groups ≥45 years were at risk for BCTB, with those ≥65 years old having 2.8 times higher risk. Other factors significantly associated with TB were: previous TB treatment, self-report of diabetes mellitus, and residing in an urban setting. Smoking and sex were analyzed as interacting variables, showing that men with more than five pack-years smoking history were 3.5 times more at risk (aOR = 3.5, 95% CI: 1.9−6.3) while women smokers had twice risk than non-smokers. Surrogate indicators for lower socioeconomic status, such as recipients of the Philippine government’s conditional cash transfer program for the poorest of the poor (called *Pantawid Pamilyang Pilipino Program*, or 4Ps) and absence of health insurance coverage, were 1.6–1.8 times more likely to have TB. Related surrogate indicators, which were analyzed as interacting variables, showed that households without a refrigerator had 1.7 times the risk of TB, irrespective of household size.

## Discussion

The estimated burden of bacteriologically confirmed pulmonary TB in the Philippines in 2016 was 1,159 per 100,000 adult population, indicating an unacceptably high number of people who are not diagnosed with or reported to have TB. This estimate, when projected to all ages and all forms of TB, translates to approximately 1 million Filipinos with TB. In contrast, in 2016, the NTP notified only 330,000 TB cases [15] − not all of which were bacteriologically confirmed − equivalent to an estimated P:N ratio of 3.1 in 2016. In addition, only half of survey participants who claimed to be on TB treatment at the time of the survey were found in the national TB registry. Those reported to the TB registry were mostly treated by the public sector, underscoring the low engagement of private providers in TB case notification. At the time of the survey, the implementing rules and regulations for mandatory notification of TB to the national TB registry, as provided by the Comprehensive TB Elimination Plan Act (Republic Act 10767), had not yet been fully developed. Other reasons for under-reporting in the country’s TB registry are: insufficient systematic TB screening and case detection, particularly among high-risk groups; limited contact tracing efforts; inadequate engagement with community-based organizations, non-governmental organizations, and the private sector including private hospitals and workplace initiatives; under-utilization of Xpert MTB/RIF services; lack of human resources and time to encode TB cases into the Integrated TB Information System of the country; lack of computer hardware and/or internet connectivity, which deter or delay reporting; and pervasive service access issues such as geographical and financial barriers and inappropriate health-seeking behaviour [16].

The high prevalence of TB in the Philippines is multifactorial. Globally, the key social determinants of TB epidemiology are global social inequalities, population mobility, and rapid urbanisation and population growth [17]. Although poverty rates in the Philippines have slightly declined through the years − poverty incidence of 21.6% in 2015 compared to 25.8% in 2014 − marked inequities persist, *e*.*g*., poverty incidence rates in 2015 of 40.8% for farmers and 36.9% for fishermen [18]. Rapid urbanisation has been associated with a rise in slum housing, with 43.5% of the population living in slum dwellings in 2016 compared to 38.3% in 2014 [19]. The higher prevalence rates seen in the urban National Capital Region (stratum 1 in this survey) and in survey clusters located in urban slums suggest that crowded living conditions fuel TB transmission.

On the demand side of health care, the healthcare seeking behaviour of symptomatic individuals remained low. Self-medication remains a prevalent practice in the country, with 43.4% reporting this action in the 2007 survey and 40.1% in the 2016 survey. However, there was a higher proportion of survey participants who took no action for symptoms suggestive of TB in the 2016 survey (41%, vs. 25% in 2007), while the proportion consulting a healthcare provider decreased (19% in 2016 vs. 32% in 2007) [20]. In both surveys, the prevailing reasons for non-consultation were perceived triviality of symptoms, costs for travel to health facilities, and missed work or school days, suggesting the need for improving social and behavior change communications and patient-centered care to address access issues, including significant out-of-pocket issues related to TB care in the country.

On the supply side, reliance on passive case detection using poorly sensitive diagnostic tools such as light microscopy likely contributed to delayed or missed case detection and treatment. Using a combination of a new rapid highly sensitive and specific molecular test (Xpert MTB/RIF), an improved DSSM with LED fluorescent microscopy, digital chest X-rays and traditional symptom screening, the survey detected more than the expected number of TB cases. Screening for TB using symptoms of cough and haemoptysis alone would have missed two-thirds of bacteriologically confirmed cases. Chest X-ray screening detected almost all PTB cases, with the number needed to screen to detect one TB case at 100. Using DSSM alone for diagnosis missed two-thirds of bacteriologically confirmed cases. Although culture remains the reference standard for TB diagnosis, Xpert MTB/RIF detected 1.7 times more TB cases than culture under field survey conditions. This suggests that a combination of community-based active case detection and intensive case finding among high-risk groups (e.g., particularly male smokers, those with diabetes mellitus, the elderly, poor households, and urban dwellers) and healthcare settings such as hospitals and private clinics with chest X-ray, screening for TB symptoms, and fairly rapid molecular diagnostic tests such as Xpert MTB/RIF could significantly ramp up case finding and treatment. These expanded case-finding and treatment efforts should be coupled with mandatory notification of TB cases, patient-centered treatment and support, and contact tracing.

Overall, the 2016 NTPS survey provided nationally representative, precise, and reliable estimates of the prevalence of TB in the country. Multiple layers of external monitoring by the WHO team, a steering committee led by the NTP that regularly monitored survey operations, and internal quality management procedures of the project team ensured that the survey was conducted according to internationally accepted standards. On the other hand, amongst the limitations of the survey were the 76% participation rate (vs. the target of 85%) as well as the relatively low rates of chest X-ray participation (88%) and sputum submission (87%). The lower-than-expected participation rate was due to a number of factors: the campaign period in the run-up to the local elections in May 2016, work or school priorities of the eligible population, several tropical cyclones, and immunization campaigns prioritized by the Department of Health. To address some of the challenges, we extended survey hours to the evenings and weekends and/or surveys days in several clusters. Moreover, missing data were accounted for in the estimation of prevalence through multiple imputation analysis.

Despite the lower-than-expected sputum submission rate, the high volume of sputum specimens submitted to only six reference laboratories led to heavy workloads in the laboratories. The paucibacillary nature of specimens, logistics and cold storage during specimen transportation, and laboratory workload may have impacted on culture performance, but laboratory performance quality indicators suggest overall acceptable performance (4.1% contamination rate and 89.0%% culture-positive among smear-positive samples).

The addition of Xpert MTB/RIF to the 2016 NTPS strengthened the detection of prevalent TB cases. However, it should be noted that there were 238 BCTB cases who were positive by Xpert alone. After the survey was completed, a 2017 study of the accuracy of Xpert MTB/RIF showed that its specificity was lower at 92.7% (95% CI: 82.4−98.0%) for those who reported TB in the past two or less years [21]. Given that 71 (29.8%) of the 238 reported a history of TB of ≤5 years, it is possible that there was some over-diagnosis of TB, with 35 (49.3%) of the 71 having a ‘very low’ Xpert semi-quantitative result. On the other hand, amongst the 222 of the 238 who also had a chest X-ray, the vast majority (204, 91.9%) had findings highly suggestive of TB as confirmed by the Diagnostic and Medical Panel.

Lastly, another limitation was the exclusion of HIV serostatus in the survey. In 2015, during survey protocol preparation, the reported incidence rate of TB/HIV was 4.3 per 100,000 [3]. At this level, the sample size and sampling requirements as well as the logistics of HIV pre-test counseling and testing exceeded the available funds and scope of the 2016 NTPS.

## Conclusion and recommendations

The 2016 nationally representative survey showed that TB remains a significant health burden in the Philippines with no evidence of decline in smear and culture positive TB compared to the 2007 survey. Reasons for the high TB burden are multifactorial, hence eliminating TB must be a national and multi-sectoral priority.

In alignment with the latest WHO guidance [22], systematic screening of subpopulations with structural risk factors using Xpert MTB/RIF should be prioritized specifically amongst men, older age groups, the poor and urban dwellers, those previously treated for TB, smokers, and those with diabetes mellitus. Engagement with communities, non-governmental organizations, and the private establishments and work places, will be critical in ramping up active and intensive case finding efforts. While TB culture remains the reference standard for diagnosis and is required for drug susceptibility testing, the rapid turnaround of results and accuracy of Xpert MTB/RIF can potentially decrease pre-treatment loss to follow-up, allow timely initiation of treatment, almost triple the TB cases detected compared to DSSM, and identify MDR-TB cases early. To ramp up case finding and treatment, laboratory capacity for Xpert testing should be expanded and decentralized and the supply chain management system should be strengthened to ensure a steady supply of Xpert cartridges and anti-TB drugs.

Addressing behavioural barriers to healthcare-seeking is central to finding undiagnosed people with TB, through improved social and behavior change interventions and patient-centered care. Innovations and implementation research are needed to strengthen behavior change technology and to support access to care strategies. Social and financial risk protection should also be priorities as the Philippine government moves towards implementation of the Universal Health Care Law.

Low notification rates of TB cases by private providers may be addressed by developing user-friendly applications for reporting to the national registry, creating social contracts with the private sector and/or civil society groups for upscaling case detection and care services, and exploring approaches to overcome barriers such as stigma and lack of confidence in the public sector. Overall, eliminating TB will require comprehensive and sustained poverty alleviation efforts and multi-sectoral partnership at the national and local levels.

## Supporting information

S1 Table Case definition algorithm used by the Diagnostic and Medical Panel. (DOCX)
