# Supplementary material for: High TB burden and low notification rates in the Philippines: The 2016 national TB prevalence survey
Source: PLoS One. 2021 Jun 4;16(6):e0252240. doi: 10.1371/journal.pone.0252240 (PMC8177416; doi:10.1371/journal.pone.0252240)
Supplement: S1 Table — (DOCX) [file pone.0252240.s001.docx]

**S1 Table. Case definition algorithm used by the Diagnostic and Medical Panel.**

| **Xpert result** | **DSSM result** | **Culture (MTB)** | **CXR central reading** | **Survey case classification** | **Category^a^** |
| --- | --- | --- | --- | --- | --- |
| Positive | Positive | Positive (any) | Any | Case | S+B+ |
| Positive | Positive | Negative/NTM/ Contamination | Any | Case | S+B+ |
| Positive | Negative | Positive (any) | Any | Case | S-B+ |
| Positive | Negative | Negative | Any | Case | S-B+ |
| Positive | NA | NA | Any | Case | S? B+ |
| Negative or Invalid/NA | Positive | Positive (any) | Any | Case | S+B+ |
| Negative or Invalid/NA | Negative | Positive (strong)^b^ | Any | Case | S-B+ |
| Negative or Invalid/NA | Negative | Positive (weak)^c^ | Suggestive | Case | S-B+ |
| Negative or Invalid/NA | Negative | Positive (weak) ^c^ | Normal/not suggestive | Non-case^d^ | S-B+ |
| Negative or Invalid/NA | Negative | Positive (weak) ^c^ | NA | Non-case^d^ | S-B+ |

^a^ S+: smear positive, S-: smear negative, B+: bacteriologically positive, B-: bacteriologically negative

^b^ Strong culture: ≥ 10 colonies

^c^ Weak culture: 1─9 colonies

^d^ Equivocal cases deemed by the Diagnostic and Medical Panel as non-cases when the culture is weakly positive and other symptoms and signs (chest X-ray, smear, and culture) are negative or not done.
